# Ischemic stroke and transient ischemic attack associated with neuroborreliosis: a Danish retrospective cohort

**DOI:** 10.1007/s00702-025-03087-8

**Published:** 2026-01-16

**Authors:** Al-Hasan Hussein Dos, Anders Hougaard, Rolf Munkholm Jensen, David Scheie, Christian Stenør

**Affiliations:** 1 Department of Neurology, Herlev and Gentofte Hospital, Borgmester Ib Juuls Vej 1, 2730 Herlev, Denmark; 2 Department of Clinical Medicine, Faculty of Health and Medical Sciences, University of Copenhagen, Copenhagen, Denmark; 3 Department of Radiology, Herlev and Gentofte Hospital, Herlev, Denmark; 4 Department of Pathology, Rigshospitalet, Copenhagen, Denmark

**Keywords:** Borreliosis, HR-VWI, Denmark, Pathology, Vasculitis, Lumbar puncture, *Borrelia burgdorferi* sensu lato

## Abstract

Central nervous system (CNS) vasculitis and stroke are rare manifestations of neuroborreliosis (NB). We aimed to describe the clinical course, paraclinical findings, treatments, and outcomes of patients with ischemic stroke and transient ischemic attack (TIA) associated with NB from a comprehensive patient cohort and compare these findings to existing literature. We retrospectively analyzed data from 3841 patients who underwent intrathecal *Borrelia burgdorferi* sensu lato antibody index testing at Herlev and Gentofte Hospital between 2016 and 2024. Patients who fulfilled the EFNS criteria for definite or possible NB and had clinical and/or radiological evidence of stroke or transient ischemic attack were included. In total, 413 patients fulfilled criteria for NB. Twelve adults and one child fulfilled our criteria for NB-associated stroke or TIA. Seven out of 13 patients presented with gait difficulty and 4/13 with upper motor neuron signs. Mean cerebrospinal fluid (CSF) glucose and CSF/blood glucose ratio were significantly lower compared to NB patients without cerebrovascular events in our cohort. Seven of 13 presented with cognitive symptoms prior to stroke. Twelve out of 13 had magnetic resonance imaging-verified brain infarctions. One patient died; autopsy confirmed lymphocytic vasculitis. High resolution vessel wall imaging, when performed (*n* = 3), showed concentric vessel wall enhancement. Cerebrovascular manifestations of NB may present acutely with infarction or with chronic prodromal symptoms preceding stroke or transient ischemic attack. High resolution vessel wall imaging appears to be a promising noninvasive tool, potentially enabling earlier diagnosis and facilitating targeted treatment research.

**Supplementary Information:**

The online version contains supplementary material available at 10.1007/s00702-025-03087-8.

## Background

Neuroborreliosis (NB) is a neurological manifestation of Lyme borreliosis, the most common tick-borne infection in Europe and North America, caused by *Borrelia burgdorferi sensu lato complex (Bbsl)* (Kullberg et al. 2020; Rauer et al. 2020). In endemic regions, up to 10–15% of patients with untreated Borrelia infection develop neurological symptoms, which can include meningitis, cranial neuritis, or radiculitis (Kullberg et al. 2020; Rauer et al. 2020). Severe cerebrovascular complications, including central nervous system (CNS) vasculitis, occur more rarely (Winter et al. 2024; Zajkowska et al. 2015; Garkowski et al. 2017; Bajons et al. 2023). The pathophysiology of cerebrovascular manifestations in NB is complex, but vasculitis is considered the primary mechanism (Winter et al. 2024; Oksi et al. 1996; Miklossy et al. 1990; Monteventi et al. 2018; Zajkowska et al. 2015; Back and Grünig 2016). Thus, autopsy and brain biopsy studies have demonstrated perivascular inflammation and lymphocytic vasculitis of the meningeal and parenchymal vessel walls (Winter et al. 2024; Oksi et al. 1996; Miklossy et al. 1990; Monteventi et al. 2018; Zajkowska et al. 2015; Back and Grünig 2016). These changes can cause ischemic or hemorrhagic stroke, transient ischemic attacks (TIA), or, in rare cases, cerebral venous sinus thrombosis (Garkowski et al. 2017; Zajkowska et al. 2015).

Traditional imaging methods for detection of NB-associated vasculitis include digital subtraction angiography (DSA), magnetic resonance imaging-angiography (MRA) and computed tomography-angiography (CTA) (Zajkowska et al. 2015; Wasserman et al. 2001; Ghorishi et al. 2023). In recent years, magnetic resonance imaging with high-resolution vessel wall imaging (HRVWI-MRI) has emerged as a promising tool for detecting vasculitis (Park et al. 2017; Ferlini et al. 2022; Sundaram et al.^2021^). However, the validity of this technique for reliably identifying vasculitis has been questioned (Park et al. 2017; Pascarella et al. 2023).

Antibiotic treatment with doxycycline or ceftriaxone is often reported to result in the recovery of neurological deficits and a generally favorable outcome (Mygland et al. 2010). Corticosteroids and, in some cases, cyclophosphamide are also frequently used, but their efficacy lacks support from randomized trials (Bajons et al. 2023; Komdeur 2001; Rauer et al. 2020).

The primary aim of this retrospective cohort study was to estimate the incidence and clinical phenotype of NB-associated stroke and TIA. Secondary aims were to compare cerebrospinal fluid (CSF) and blood test results between NB patients with and without cerebrovascular events, and to summarize imaging findings and treatment outcomes.

## Methods

### Inclusion process and case definitions

As described in a previous publication, information was retrospectively gathered from electronic charts of 3841 patients who underwent a CSF *Borrelia burgdorferi* (*Bb*) antibody index (*Bb*-AI) test (see below) at the Department of Microbiology at Herlev and Gentofte Hospital (HGH), Capital Region of Denmark, between January 2016 and January 2024 (Dos et al. 2025). This department services both HGH and North Zealand Hospital (NZH). Data regarding admission date, tick exposure, clinical symptoms, CSF parameters and *Bb* serology were collected and stored in a REDCap database (v.14.1.2) (Harris et al. 2009). Adults were defined as individuals aged ≥ 18 years.

Definite and possible NB patients were included and classified per the guidelines of the European Federation of the Neurological Society’s (EFNS) criteria (Mygland et al. 2010).

In accordance with previous studies, fulfillment of three criteria was required for defining a case as NB-associated stroke or TIA (Garkowski et al. 2017; Winter et al. 2024): (1) Fulfillment of the EFNS criteria for definite or possible NB (Mygland et al. 2010), (2) Clinical and/or radiological evidence of ischemic stroke, TIA, or vasculitis, and (3) No alternative stroke or TIA etiology identified on routine clinical work-up (e.g., no evidence of other neuroinfections, atrial fibrillation, or clinically significant carotid stenosis). We applied the time-based definition of TIA as a focal neurological dysfunction of presumed vascular origin with a duration < 24 h (Degan et al. 2017).

CSF data were compared between NB patients with and without cerebrovascular manifestations within the cohort. Since 12 out of 13 of the NB patients with cerebrovascular manifestations were adults, the comparison was exclusively performed between the group with ischemic stroke and TIA and the adult subgroup of NB patients without cerebrovascular events.

We estimated all ischemic stroke cases during our study period based on incidence data from the Danish Stroke Registry reports (Danish Stroke Registry 2019).

### *Borrelia burgdorferi* serology

All analyses for *Bb* antibodies were conducted in accordance with the Danish guidelines for diagnosing LB (Dessau et al. 2014).

### Serum *Borrelia burgdorferi* antibodies

*Bb* antibodies in serum were assessed using LIAISON *Borrelia* Immunoglobulin G (IgG)/*Borrelia* Immunoglobulin M (IgM) Quant (DiaSorin, Italy). Since May 2017 only *Bb* IgG values were measured.

### Intrathecal *Bb* antibody test

*Bb*-AI IgG and IgM were measured using a second-generation flagella antigen-based capture enzyme immunoassay (IDEIA Lyme Neuroborreliosis, Oxoid, Hampshire, UK) (Hansen and Lebech 1991). This method involves a capture enzyme immunoassay (EIA), which determines the ratio of anti-Bb-specific antibodies to total antibodies of the same class in both CSF and serum. An elevated *Bb*-AI of ≥ 0.3 was considered positive.

### Statistics

All statistical analyses were performed with R (version 2024.4.0.735) (Posit team 2024). Continuous variables were assessed for normality with histograms, and statistical tests were applied accordingly: normally distributed continuous variables were compared using the independent two-sample t test, while non-normally distributed (skewed) data were analyzed using the Mann–Whitney U test. A p value < 0.05 was considered statistically significant. Data are presented as mean ± standard deviation (SD) for normally distributed variables and as median (range) for skewed variables. Analysis of covariance (ANCOVA) was employed to evaluate the relationship between symptom duration and CSF glucose in NB patients with ischemic stroke or TIA.

### Ethical approval

The study was approved by the Danish Health Authorities according to the Danish Health Act paragraph 46 Sect. 2 and paragraph 48 Sect. 1 (J-23033262). Ethical approval and consent from individual patients are not required for this type of study according to Danish legislation.

## Results

### Baseline characteristics

Of the 3841 patients who underwent a *Bb*-AI test between January 2016 and January 2024, a total of 413 patients fulfilled EFNS criteria for definite or possible NB. Out of those, 267 were adults and 146 were children. Thirteen of those patients, 12 adults and one child, were classified as having NB-associated stroke or TIA based on our inclusion criteria. Five out of the 13 patients (patients no. 1, 2, 6, 8, and 12, see Tables 1, 2, 3) were initially admitted with stroke or TIA before evaluation for NB, while the rest were initially diagnosed with or evaluated for NB before their stroke or TIA. Eleven patients fulfilled the EFNS criteria for definite NB, while two patients (patients no. 12 and no. 5, see Tables 1, 2, 3) fulfilled the criteria for possible NB and for definite late NB with polyneuropathy, respectively.

**Table 1:** Baseline characteristics of 13 neuroborreliosis patients with cerebrovascular manifestations from the capital region of Denmark, 2016–2024

Patient no.	Gender (m/f)	Age (y)	Cerebrovascular risk factors	Symptom duration (mo.)	History of tick bite/rash	Relevant treatment (treatment length)	Outcome (time of follow-up, months after the initial stroke or TIA)^a^
1	f	39	N/A	29	Yes/no	Ceft 4 g x 1 (1D), dox 100 mg x 2 (3 W), ASA 75mgx1 (N/A)	Partial recovery. Improvement in gait, urinary urge and retention. Continued cognitive issues, especially fatigue but improved(10)
2	m	22	N/A	60	No/no	Ceft 2 g x 1 (2 W), dox 100 mg x 2 (2 W)^b^, clopidogrel 75 mg x 1 (N/A), pred 150 mg x 1 (2 W)^c^	Left hemiplegia, gait and balance difficulty. Can study (97)
3	m	69	Smoking (p)	5	Yes/no	Ceft 2 g x 1 (2 W), dox 100 mg x 2 (2 W), ASA + clopidogrel 75 mg x 1 (N/A), pred 100 mg x 1 (4 W)^d^, methylprednisolone 1000 mg x 1 (4D), methylprednisolone 500 mg x 1 (4D)	Dead (3)
4	f	56	Smoking (A)	14.5	No/yes	Ceft 4g x 1 (4D), dox 200 mg x 2 (2 W), ASA + clopidogrel 75 mg x 1 (N/A)	Improvement in fatigue, weight and gait. Improved left leg paresis. Improved concentration (4)
5	f	71	HT,	72	No/yes	Penicillin 2MIU x3 (17D), dox 100 mg x 2 (2 W), ASA + clopidogrel 75 mg x 1 (N/A)	Continued multiple strokes, low level of consciousness, and no speech (34)
6	m	37	Smoking (p)	2	No/no	Ceft 2g x 1 (10D), ASA + clopidogrel 75 mg x 1 (N/A)	Improved but persistent fatigue and memory difficulty (6)
7	f	12	N/A	1	Yes/no	Ceft 4 g x 1 (4D), dox 100 mg x 2 (10D), ASA 75 mg x 1 (365D)	Complete recovery (14).
8	m	78	Smoking (p)	10	No/no	Ceft 2 g x 1 (2 W), pred 50mgx1 (N/A)^	Continued gait difficulty, memory issues, and diplopia (42). Suspected concomitant neurosarcoidosis (NS)
9	f	76	HT, smoking (p)	0	No/no	Ceft 4 g x 1 (2D)^e^, Ceft 2 g x 1 (5D), dox 100 mg x 2 (10D)	Memory difficulty with gradual improvement (12)
10	m	54	Smoking (A), HC	0	No/no	Dox 100 mg x 2 (2 W), ASA + clopidogrel 75 mg x 1 (N/A)	Subtle cognitive issues with memory, concentration, speech, and initiative (5)
11	f	71	N/A	4	Yes/yes	Ceft 4 g x 1 (1D), dox 100 mg x 2 (2 W), ASA 75 mg x 1 (90D)	Overall improvement but still dizziness (2)
12	m	52	Smoking (p)	0	N/A/N/A	Dox 100 mg x 2 (3D), Ceft (2D), clopidogrel (6D), apixaban (N/A)	Near-complete recovery with only discrete aphasia (3)
13	m	79	HC, smoking (p), DM2	12	No/no	Ceft 2g x 1 (5D), dox 100 mg x 2 (14D)	Improvement in gait, memory and fatigue but still mildly present (27)

m (male), f (female), y (years), mo. (months), TIA (transient ischemic attack), N/A (not applicable), p (previous), A (active), HT (hypertension), HC (hypercholesterolemia), ceft (ceftriaxone), dox (doxycycline), ASA (acetylsalicylic acid), D (days), W (weeks), MIU (million units), DM2 (diabetes mellitus type 2)

^a^For every patient except no. three, five and seven, the follow-up was done in the neurological outpatient clinic. For patient three he was re-admitted and eventually succumbed to his neurological deficits in the neurological department. Final follow-up on patient five was in the Department of Internal Medicine. For patient seven follow-up was performed in the pediatric out-patient clinic

^b^The patient was later retreated with ceftriaxone 2 g x 1 for 2 weeks and doxycycline 100 mg x 2 for 2 weeks

^c^Tapering to 50 mg x 1

^d^Tapering

^e^Tapering to 2 g x 1

**Table 2:** Clinical presentation, findings and imaging of 13 neuroborreliosis patients with cerebrovascular manifestations from the capital region of Denmark, 2016–2024

Patient no.	Reported symptoms	Objective findings	Imaging
1	Gradual neck and arm pain, gait and coordination difficulty, prickling sensation, headache, urinary urge. Later admission with transient acute speech impairment	Spastic paraparesis, hyperreflexia, gait difficulty, dysarthria, urinary retention. Aphasia on later admission	(1) MRI-NA: meningeal enhancement of the spinal medulla, cranial nerves and brainstem (Fig. 1), (2) HRVWI-MRI + C: concentric vessel wall enhancement and narrowing of basilar artery, left SCA and VA (Fig. 1), (3) CTA: occlusion of the left VA
2	Initial wake-up symptoms of left sided arm and leg weakness and facial paralysis Later admission with confusion, amnesia, déjà vu	Spastic hemiparesis, hemisensory deficit, hyperreflexia, bilateral Babinski	(1) Brain MRI: Infarction in the right centrum semiovale (Fig. 2), (2) Brain MRI: Infarction in the left hippocampus (Fig. 2), (3) Brain MRI + C longitudinal perivascular enhancement of the BA and contrast enhancement of CN V, VII, VIII (Fig. 2), (4) MRA and DSA: no focal arterial stenosis, (5) Brain MRI: pontine infarction (Fig. 2) (6) TTE: normal
3	Gradual confusion, aphasia, amnesia, disorientation, behavioral changes, fatigue, coordination problems, inattention	Disorientation, decreased short term memory, progressive hemiparesis, coma, and death.	(1) Brain MRI + C: Bilateral thalamic infarction (suppl Fig. 2), (2) HRVWI-MRI + C: concentric enhancement of the PCA, BA and VA (Fig. 2), (3) Brain MRI: infarction in the right thalamus, pons and cerebellum (Fig. 2), 4)¹⁸F-FDG-PET-CT: hypometabolism in the left cerebrum and right cerebellum and hypermetabolism in the left mesial temporal lobe (suppl. Figure 3)
4	Gradual gait difficulty, urge, urinary retention, impaired concentration, fatigue, 12 kg weight loss^a^	Dysarthria, spastic atactic tetraparesis, bilateral Babinski, truncal instability	(1) MRI-NA + C: Infarction in the pons, contrast enhancement of leptomeninges around temporal lobes, pons, mesencephalon, CN, spinal roots (Fig. 5) (2) HRVWI-MRI + C + MRA: Concentric enhancement of the VA, BA, SCA and left MCA (M1 and proximal M2, where there is stenosis in the time-of-flight sequences) (Fig. 5)
5	Gradual gait difficulty, dysesthesia, hypesthesia, leg pain. Later admission with acute aphasia and hemiparesis	Acrodermatitis chronica atrophicans, dysesthesia in her legs, hemiparesis, aphasia	(1) Multiple brain MRIs: Multiple infarctions of varying age bilaterally and in the anterior and posterior fossa, (2) Brain MRI + C: no enhancement (months after treatment)
6	Gradual neck pain, headache, nausea, vomiting	Normal	(1) MRA: right pons infarction, reduced flow void in right VA, (2)CTA: thrombosis in the right VA, (3) Brain MRI + C: No contrast in right VA, reduced BA lumen
7	Initial gradual headache, fatigue, nausea, vomiting. Later admission with acute transient confusion and speech impairment	Disorientation	(1) MRA + C: cystic /chronic infarction lesion of the left internal capsule and corona radiata. Leptomeningeal enhancement bilaterally around occipital lobes and multiple cranial nerve enhancement^b^
8^c^	Initial gradual gait difficulty, fatigue, vertigo, diplopia. Later admission with acute impaired speech^d^	Dysarthria, hemiparesis, hyperreflexia, Babinski, ataxia, spastic gait	(1) Brain MRI + C: Minor bilateral cortical infarctions (confirmed on later brain MRI) and multiple nodular contrast enhancing lesions (2) Brain MRI + C: regression of nodular processes, now believed to be of NS origin, (3) Spine MRI + C: transverse myelitis, diffuse enhancement4) ¹⁸F-FDG-PET-CT: multiple nodules in the lungs, lymph nodes, muscles and sternal and costal bones
9	Gradual confusion, disorientation, nausea. Wake up symptoms with facial weakness	Unilateral peripheral facial palsy, hemiparesis, disorientation	(1) Brain MRI: diffuse bilateral cortical DWI and ADC lesions, (2) ULC: no stenosis
10^e^	Initial gradual neck, shoulder, back pain, dysesthesia in both legs, facial weakness. Later admission with acute left sided weakness in the arm and leg.	Unilateral peripheral facial palsy. Later left sided hemiparesis.	(1) CTA: no large artery occlusion / stenosis, (2) Brain MRI: Acute FLAIR positive infarction in the right lentiform nucleus, posterior internal capsula, caudate nucleus
11	Gradual fatigue, gait difficulty, memory issues, 5 kg weight loss, chronic headache, radicular pain in extremities.	Bilateral peripheral facial palsy, gait difficulty	(1) Brain MRI + C: Multiple subacute infarctions in the right thalamus and basal ganglia and left cerebellum, (2) ULC: no stenosis
12	Wake-up with confusion, memory issues, impaired speech, behavioral changes	Disorientation, aphasia, amnesia, neglect, bilateral palmomental reflex	(1) Brain MRI: multiple acute infarctions bilaterally in the frontal lobes, corpus callosum, and left basal ganglia (confirmed on later brain MRIbra), (2) CTA: normal, (3) DSA: normal
13	Gradual gait difficulty, dizziness, short-term memory issues, fatigue, 8 kg weight loss^f^	Disorientation, short-term amnesia, executive cognitive issues, wide-based gait, positive Romberg test, paratonia in the legs and echopraxia in the arms	(1) Brain MRI + C: FLAIR positive infarction in the right hippocampus and normal pressure hydrocephalus, (2) Brain MRI: Chronic infarction lesion in the right hippocampus and no sign of normal pressure hydrocephalus, (3) ¹⁸F-FDG-PET: normal, no sign of neurodegenerative activity

CTA (Computed tomography-angiography), MRI (magnetic resonance imaging), MRA (magnetic resonance imaging angiography of the cerebrum), NA (neuroaxis) DSA (digital subtraction angiography),¹⁸F-FDG-PET-CT ([^18^F]Fluorodeoxyglucose positron emissiontomography–computed tomography), TTE (transthoracic echocardiogram), HRVWI-MRI (MRI High resolution vessel wall imaging), C (contrast), MCA (middle cerebral artery), ULC (duplex ultrasound of the carotid arteries), FLAIR (fluid attenuated inversion recovery), DWI (diffusion-weighted magnetic resonance imaging), ADC (apparent diffusion coefficient), SCA (superior cerebellar artery), BA (basilar artery), VA (vertebral artery)

^a^The patient’s body-mass index at admission was 16.8

^b^Follow-up MRI with contrast a year later showed no pathological contrast enhancement

^c^It was concluded that the patient had concurrent neuroborreliosis and neuro-sarcoidosis

^d^The patient was admitted with 1–2 h lasting acute aphasia, interpreted as a transient ischemic attack (TIA)

^e^The patient was also monitored for atrial fibrillation with a 3-day Holter monitor, which did not show any abnormalities

^f^Developed over 2 months. No Weight or BMI was recorded at admission

**Table 3:** Cerebrospinal fluid and blood results of 12 neuroborreliosis patients with cerebrovascular manifestations at admission from the capital region of Denmark, 2016–2024

Patient no.	CSF cell count /µL (% mononuclear cells)	CSF total protein g/L	CSF glucose mmol/L (CSF/blood ratio)	IgG/IgM Bb AI	S-IgG AU/mL	^a^CXCL-13 /NFL ng/L	Other blood tests	Other CSF tests
1	137 (90)	5.39	0.5 (0.08)	9.7/12.4	173.9	N/A	N/A	ME^b^, cryptococcus, Mycobacteria D + R and PCR—all neg, Q-Alb 27.14, IgG index 0.91
2	55 (97)	2.01	1.3 (0.26)	9.9/22.3	> 240	109.36/2,900^c^	Syphilis antibodies (neg), heterozygote factor V leiden, positive prothrombin, neg cardiolipin ab	HSV/VZV PCR and IgG, TBE PCR, syphilis ab—all neg, IgG index 2.49, Q-alb 22.35
3	136 (98)	1.00	2.5 (0.47)	Incalculable due to very high antibody titers in both CSF and blood at 3 different sample times/0	> 240	> 500/4,060	TBE IgM/IgG, Rickettsia ab, cryptococcus antigen test, syphilis ab—all neg	CSF IgG and PCR of HSV/VZV, ME, cryptococcal antigen test, Neoehrlichia ,*Borrelia Miyamotoi* and *Bbsl PCR*, mycoplasma PCR, enterovirus PCR and microbiome 16 S and 18 S PCR—all neg, Q-Alb 5.35, IgG index 2.96
4	105 (98)	4.09	1.7 (0.32)	28.7/2.80	> 240	> 500/13,892	Cryptococcus Ag neg, syphilis ab neg,	ME and IgG, syphilis antibodies and Cryptococcus Ag—all neg, Q-Alb 30.81, IgG index 0.82
5	1	0.26	2.7 (0.58)	12.7/0	> 240	N/A/N/A	Syphilis Ab neg	Syphilis AI Ab neg
6	832 (68)	3.72	0.7 (0.10)	17.8/0.2	232	N/A/18,948	TBE IgM/IgG, syphilis ab, ANA/ANCA, anti-SSA/SSB ab, cardiolipin Ab, beta-2-glykoprotein ab—all neg	Q-Alb 45.1, IgG index 1.21, HSV/VZV PCR neg
7	107 (97)	1.97	2.0 (0.37)	7.0/2.8	234.1	N/A/N/A	EBV and CMV IgM/IgG neg/pos, toxoplasmosis IgG/IgM neg,	ME neg, Q-alb 30, IgG index 1.08
8	35 (99)	1.90	2.9 (0.40)	6.6/6.8	43.4	N/A/16,321	Quantiferon test neg, IL-2R 692, ACE 30	MEP^†^, syphilis antibodies, toxoplasmosis PCR, microbiome S PCR, taenia species PCR—all neg, Q-Alb 26.90, IgG index 0.88
9	207 (100)	2.39	3.5 (0.50)	0/39.4	176.0	N/A/N/A	Na^+^ 119, TBE IgM/IgG neg	HSV/VZV IgG and PCR, TBE IgG/IgM—allneg
10	127 (96)	1.53	2.8 (0.46)	0/11.6	238.8	N/A/N/A	N/A	N/A
11	104 (91)	1.68	2.4 (0.38)	5.1/1.1	183.8	N/A/N/A	N/A	TBE PCR neg, HSV/VZV PCR neg, enterovirus PCR neg
12	2	0.38	3.8 (0.64)	0.8/0	> 240	N/A/N/A	Syphillis Ab neg	HSV/VZV PCR neg
13	96 (99)	1.22	3.1 (0.44)	Incalculable due to very high antibody titers in both CSF and blood/11.9	> 240	N/A/1,790	Syphillis Ab neg, TBE IgM and IgG neg	MEP neg, HSV/VZV IgG neg

neg (negative), CSF (cerebrospinal fluid), mo. (months after initial test), D (days after initial test), IgG/M (immunoglobulin G/M), N/A (not applicable), PCR (polymerase chain reaction), *Bb* (*Borrelia burgdorferi)*,* Bbsl (Borrelia burgdorferi sensu lato)* AI (antibody index), S-IgG (serum immunoglobulin G), CXCL-13 (chemokine ligand 13), NFL (neurofilament light chain), TBE (tick borne encephalitis), HSV (herpes simplex virus), VZV (varicella zoster), ab (antibodies), Ag (antigen) IL-2R (interleukin-2 receptor), ACE (angiotensin converting enzyme), Na+ (sodium), EBV (Epstein-Barr virus), CMV (cytomegalovirus), ANA (antinuclear antibody), ANCA (antineutrophil cytoplasmic antibodies), anti-SSA/SSB (anti-sjogren’s-syndrome-erlated antigen A/B)

^a^CXCL-13 / NFL / IgG index / Q-alb were only applicable if tested early in the course or while disease is active (otherwise N/A)

^b^Biofire FilmArray^®^ Meningitis Encephalitis Panel (MEP) (Biofire Diagnostics, Salt Lake City, UT) (used at Herlev Hospital until 2022) and QIAstat-Dx Meningitis/Encephalitis (ME) Panel (QIAGEN, Germany) (used at Herlev Hospital from 2022 and onwards) test PCR for *Streptococcus pneumoniae*, *Neisseria meningitidis*, *Listeria monocytogenes*, Haemophilus influenzae, *Escherichia coli* K1, *Streptoccoccus pyogenes*, Enterovirus, HSV-1/2, Human parechovirus, VZV, Mycoplasma pneumoniae, Crytpococcus gattii and neoformans

^c^Test taken 1.5 years after treatment initiation

The clinical course of 3 out of 13 cases is detailed in the text below, while the remaining cases are summarized in Tables 1, 2 and 3. These three cases were selected to illustrate the often progressive and disabling nature of NB-associated ischemic stroke and vasculitis, as well as the varied outcomes following antibiotic treatment. Tables 1 and 2 include clinical course, tick exposure, age, comorbidity, gender, imaging results, and treatment for patients 1–13. Table 3 presents Bb serology and relevant CSF and blood test results. Supplementary Table 1 compares CSF data of the 13 NB stroke/TIA patients with the rest of the cohort.

Among 12,333 registered patients with ischemic strokes admitted to HGH and NZH during the study period, our NB patients accounted for 0.1% of cases.

Six out of the 13 patients were female (Table 1). The mean age was 55 ± 21 years.

Two out of 13 were active smokers, 4/13 previous smokers, 2/13 had hypertension (HT), 1/13 had diabetes mellitus type 2 (DM2), and two patients had hypercholesterolemia. Four patients had no vascular risk factors.

The median time to diagnosis from symptom onset was 5 months (range 0–72).

### Clinical presentation

Upper motor neuron (UMN) signs: Four out of 13 presented with UMN signs (spastic hemiparesis, Babinski sign or hyperreflexia). Two out of 13 presented with urinary problems (Table 2).

Chronic prodromal symptoms: Four out of 13 presented with chronic headache and 7/13 with cognitive issues (fatigue, disorientation, concentration problems, confusion) prior to their stroke or TIA (with a median duration of 4.5 months for either of these symptoms prior to diagnosis). Patient 2 and 12 (Table 2) presented with acute stroke symptoms, without a prodromal phase, prior to first contact with the hospital.

Three out of 13 patients presented with or were found to have peripheral facial palsy (2/3 unilateral, 1/3 bilateral) and 3/13 presented with symptoms of meningoradiculitis with or without peripheral facial palsy.

Four patients reported a tick bite, and two had a rash prior to admission. On brain imaging (ischemic changes on diffusion weighed imaging (DWI) or concentric enhancement of the arteries on HRVWI-MRI in case 1), three patients showed involvement of the posterior circulation (patient 1, 3, 6, Table 2), three had anterior circulation involvement (patient 7, 10, 12, Table 2), and six displayed both anterior and posterior circulation involvement (patient 2, 4, 5, 9, 11, and 13, Table 2).

### Cerebrospinal fluid analysis and comparison with non-vasculitis patients

Comparisons were restricted to adults: the 12 adult NB patients with cerebrovascular manifestations vs. the remaining 255 adult NB patients without cerebrovascular events. Mean CSF glucose was significantly lower in patients with cerebrovascular manifestations compared to the rest of the cohort, 2.11 ± 0.95 mmol/L (data available for all 13 patients) vs. 3.40 ± 0.94 mmol/L (*p* < 0.001, data available from 245 out of 255 patients, Supplementary Table 1). Mean CSF/blood glucose ratio was also significantly lower in patients with ischemic stroke or TIA compared to the rest of the cohort, 0.38 ± 0.16 (13/13 patients) vs. 0.53 ± 0.12 (202/255 patients) (*p* = 0.007). We found no relationship between CSF glucose and symptom duration for adult patients with cerebrovascular manifestations (ANCOVA, *p* = 0.322).

Mean protein was higher in patients with cerebrovascular manifestations compared to the rest of the cohort, 2.12 ± 1.48 g/L (13/13 patients) vs. 1.24 ± 0.77 g/L (247/255), with a trend toward statistical significance (*p* = 0.055). There was no significant difference in median WBC count of patients with cerebrovascular manifestations and the rest of the adult cohort 105µL (1-832 cells/µL; 13/13 patients) vs. 78/µL (0-1350 cells/µL; 253/255 patients) (*p* = 0.83), respectively. Both median CSF/blood albumin quotient (Q-Alb) and Immunoglobulin-G Index (IgG index) were higher in patients with cerebrovascular manifestations compared to the rest of the cohort although this was not statistically significant (*p* = 0.346 and 0.109, respectively).

Chemokine ligand 13 (CXCL-13) (reference < 20 ng/L (Knudtzen et al. 2020) was recorded early or during active disease course in 3/13 of our patients with cerebrovascular manifestations(Table 3). It was elevated in 3/3 patients, all of which had a value > 100ng / L, indicating a strongly increased concentration (Knudtzen et al. 2020). Neurofilament light chain (NFL) (ref < 723 ng/L(Vermunt et al. 2022) was recorded in 6/13 patients early or during the active disease course. All six had an elevated concentration with a median value of 8976 (range 1790–18,948) ng/L.

### Case reports

### Case/patient 1

In January 2022, a 39-year-old woman was admitted with recurrent episodes of transient speech impairment, prickling sensations on the right side of her face, and a peculiar feeling in her tongue. She was treated with thrombolysis, although brain MRI showed no diffusion restriction. However, neurologic examination revealed unexplained UMN signs, including spastic paraparesis with Babinski sign, hyperreflexia in both upper and lower extremities, gait difficulty, pseudobulbar dysarthria, and ataxia in all extremities.

**Fig. 1:**
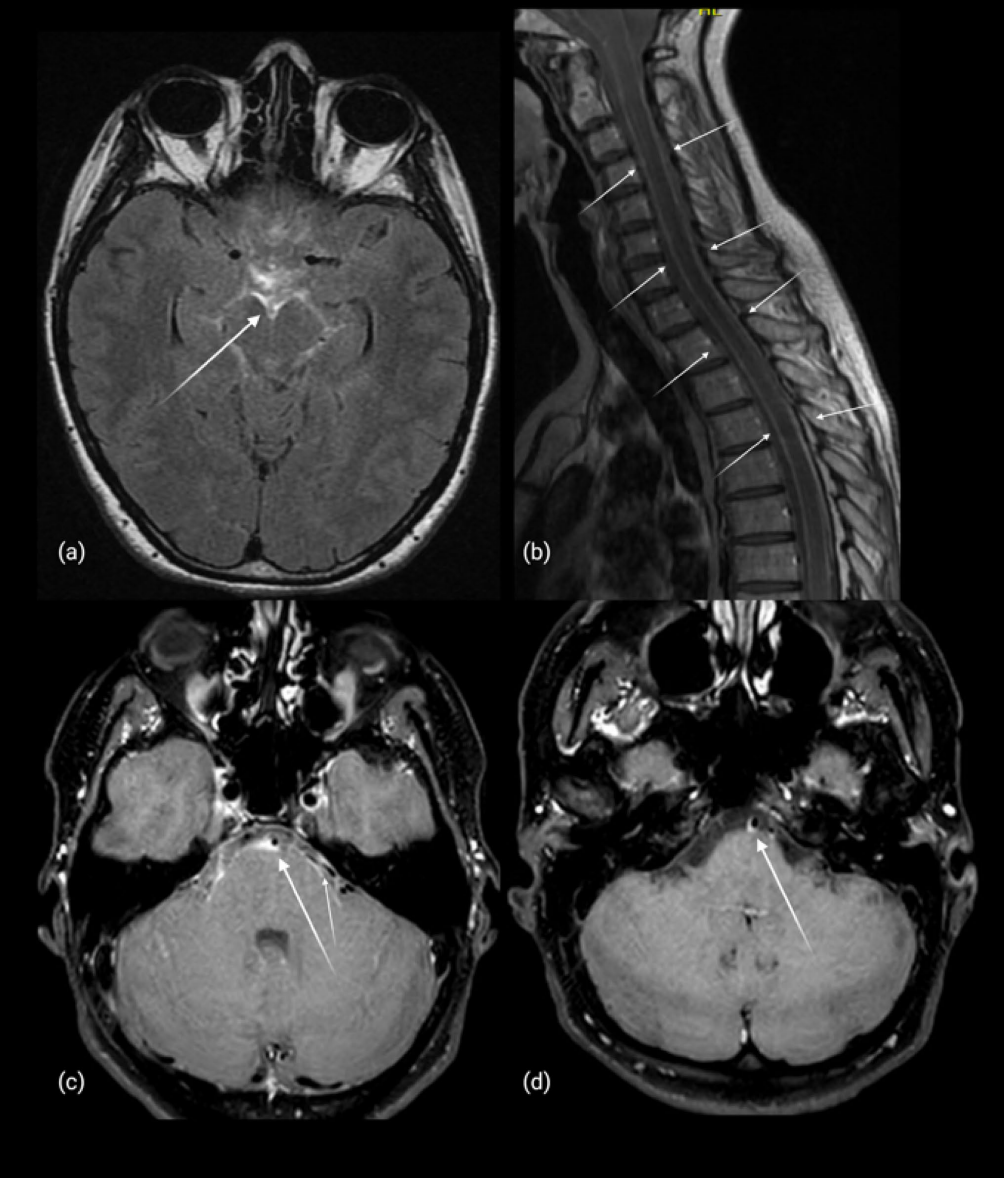
MRI of the cerebrum and neuroaxis with contrast and HRVWI-MRI sequences (2021–2022), case 1. **a** Post-contrast FLAIR leptomeningeal enhancement of the brainstem (white arrow), **b** post-contrast T1 leptomeningeal enhancement of the spinal medulla (white arrows), **c** initial HRVWI-MRI with concentric enhancement of the left SCA (short arrow) and BA (long arrow) wall and significant reduction of the lumen (white arrows). The left VA was occluded distal to PICA on HRVWI-MRI but had preserved flow voids on the first MRI (not shown), **d** HRVWI-MRI 3.5 months later with regression of the concentric enhancement around the BA and increasing lumen size (white arrow). MRI (magnetic resonance imaging), SCA (superior cerebellar artery), BA (basilar artery), VA (vertebral artery), FLAIR (fluid attenuated inversion recovery), HRVWI-MRI (MRI High resolution vessel wall imaging)

Upon reviewing her history, a prior emergency room visit in July 2020 was noted, where she presented with significant neck and left arm pain, exacerbated at night. These symptoms were attributed to a prior head trauma. By spring 2021, her condition worsened, with gait impairment, leg stiffness, intermittent prickling sensations, coordination difficulties, headaches radiating to the neck, and nighttime urinary urgency. This progression led to evaluation at an outpatient clinic in November 2021. Due to suspicion of multiple sclerosis, an MRI of the neuroaxis was ordered. Initially described as normal, upon reevaluation in January 2022, it revealed pronounced leptomeningeal enhancement of the spinal cord and brainstem (Fig. 1a, b). A lumbar puncture showed elevated protein (5.39 g/L; ref: 0.2–0.6 g/L (McCudden et al. 2017), pleocytosis (WBC 137/µL; ref: 0–4/µL, 90% mononuclear (Overview of Cerebrospinal Fluid Cytology 2018), and a very low glucose level (0.5 mmol/L; ref: 2.8–4.4 mmol/L (Leen et al. 2012). The CSF-to-blood glucose ratio was also markedly reduced (0.08; ref: 0.48–0.87 (Leen et al. 2012).

Further analysis confirmed intrathecal production of Borrelia-specific antibodies with positive Bb-AI IgM and IgG. Serum Bb IgG was also positive, with a high titer of 173.9 AU/mL (Table 3).

MRI with contrast and HRVWI-MRI showed, respectively: basal leptomeningitis with involvement of multiple cranial nerves, and concentric vessel wall enhancement of the superior cerebellar artery and basilar artery (BA) with luminal narrowing as a sign of vasculitis (Fig. 1c). There were still no ischemic changes. Ultrasound of the carotid arteries and 24-hour Holter monitor were normal.

She was initially treated with 1-day intravenous (IV) ceftriaxone therapy and later doxycycline 100 mg × 2 for 21 days. She was also treated with life-long prophylactic acetylsalicylic acid (ASA) and statins to prevent stroke (Table 1).

CSF 1 month later showed a significant improvement with a near normal CSF/blood-glucose ratio of 0.35, mononuclear leukocytes of 56 and a protein level of 1.63. MRI with contrast and HRVWI-MRI in October 2022 showed total regression of leptomeningeal enhancement and regression of the concentric enhancement around the basilar artery (BA), consistent with regressing vasculitis (Fig. 1d). In September 2023, she was readmitted with multiple minute-lasting episodes of transient left sided hemiparesis, dysarthria and dysesthesia. MRI with contrast and HRVWI-MRI showed further regression of the concentric enhancement surrounding the BA and no ischemic changes. However, CTA showed occlusion of the left vertebral artery (VA), and the episodes were attributed to cerebral hypoperfusion due to the occlusion. Her CSF, including CXCL-13 and NFL, had normalized by this point. Two years after treatment, she was able to work 10 h per week, and by 2 years and 10 months, she had increased her workload to 20 h per week and could run short distances.

#### Case/patient 2

**Fig. 2:**
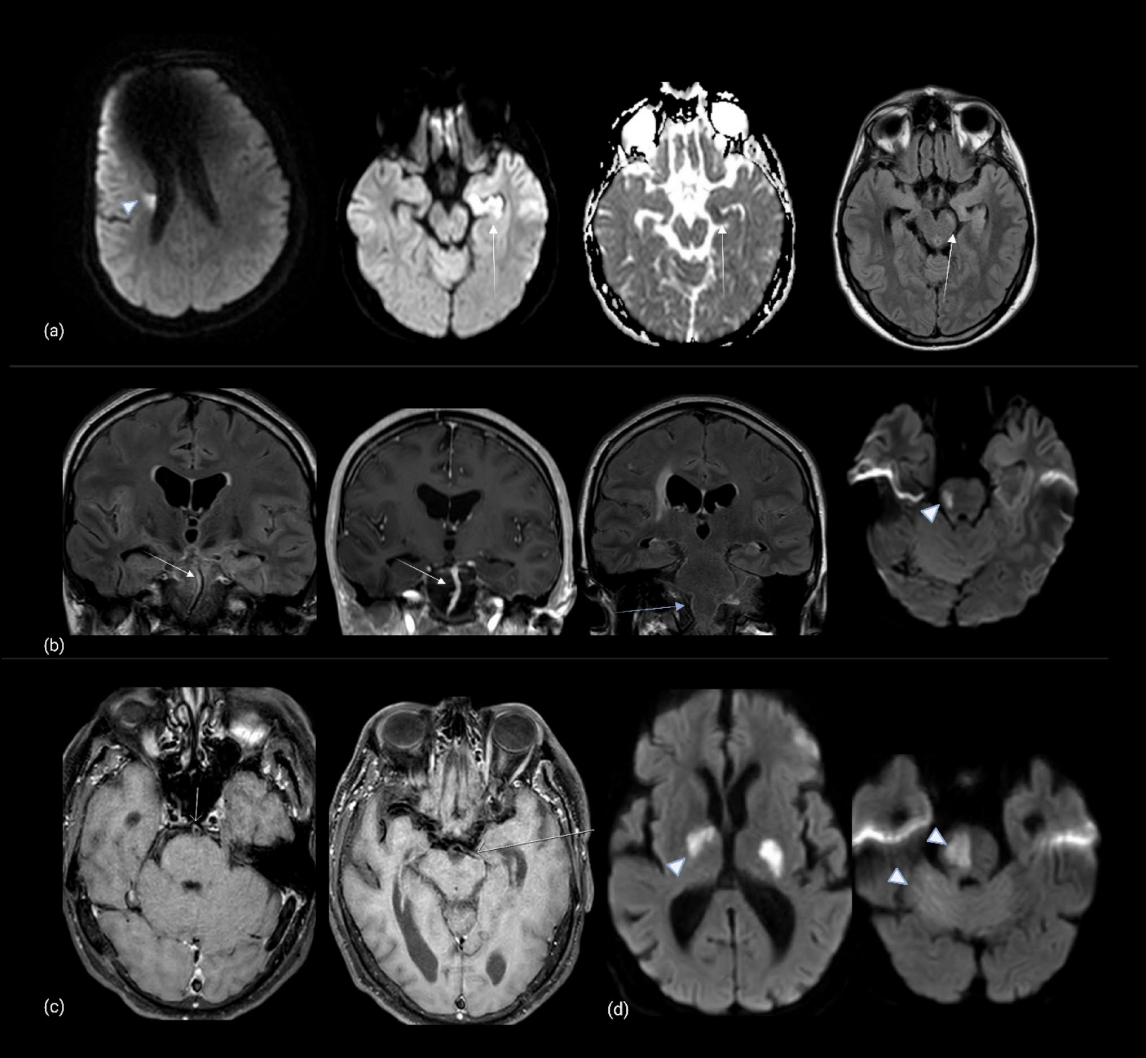
MRI of the cerebrum 2012–2023, case 2 and 3. **a** (Case 2): acute infarction in the right centrum semiovale on DWI (white arrowhead) (USA 2012), which grew in size on control MRI 12 days later (not shown, DK 2012), and acute infarction in the left temporal lobe on DWI, ADC and FLAIR (white arrows) (DK 2017), respectively. **b** Longitudinal (peri)vascular enhancement of the BA on post-contrast coronal FLAIR (long white arrow) with irregular abluminal contour on postcontrast T1 sequences (short white arrow). Appreciate the much thinner basilar lumen compared to the width of contrast enhancement. Also, leptomeningeal enhancement around the brainstem is seen (blue arrow) and cranial nerves (not shown (2017)). Acute pontine infarction on the right side on DWI (white arrowhead, 2018)). **c** (Case 3): HRVWI-MRI with concentric enhancement of the BA, PCA and left VA, respectively (white arrows). **d** Acute infarction with DWI lesions in the right thalamus, pons and cerebellum (white arrowheads). HRVWI-MRI (MRI High resolution vessel wall imaging), BA (basilar artery), PCA (posterior cerebral artery), VA (vertebral artery), BA (basilar artery), MCA (middle cerebral artery), FLAIR (fluid attenuated inversion recovery), DWI (diffusion-weighted magnetic resonance imaging), ADC (apparent diffusion coefficient), MRI (magnetic resonance imaging)

A 22-year-old man was admitted in June 2017 with acute confusion, amnesia, and déjà vu. He had a history of stroke at the age of 17 while on a trip to Los Angeles, USA, where he woke up with left hemiparesis and dysarthria. MRI of the cerebrum revealed acute and chronic infarction in the right internal capsule and left thalamus, respectively (Fig. 2a). MRA, ultrasound of the carotid arteries, and a transthoracic echocardiogram (TTE) were described as normal. Lumbar puncture in the USA revealed lymphocytic pleocytosis, but no viral or bacterial cause was found according to his discharge summary. He was discharged with prophylactic clopidogrel.

**Fig. 3:**
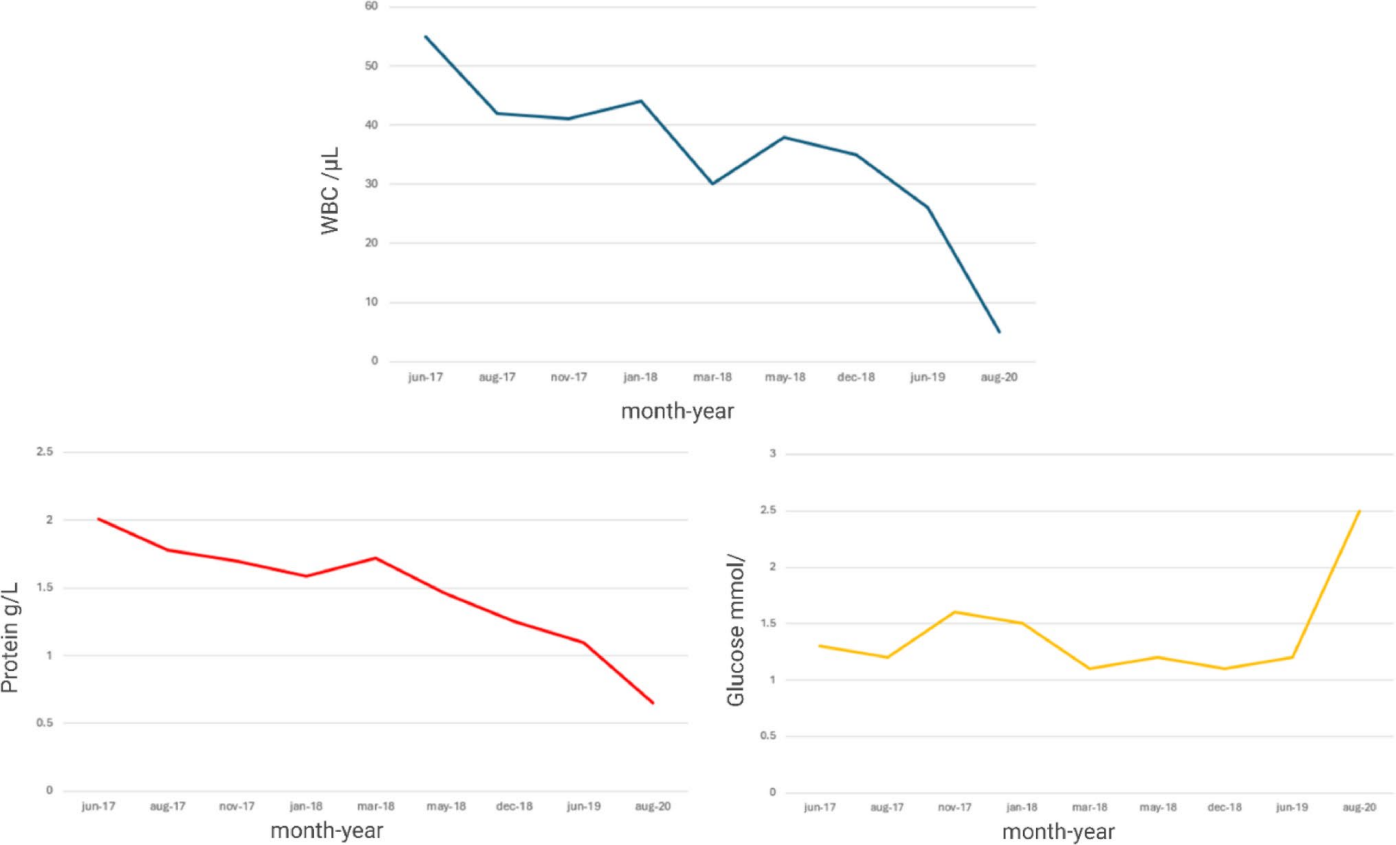
Longitudinal development of inflammatory markers in the cerebrospinal fluid of patient 2, January 2017–august 2020. CSF (cerebrospinal fluid), WBC (white blood cell)

Neurological examination, in 2017, revealed amnesia, disorientation, left spastic hemiparesis, hemisensory deficit, left hyperreflexia, and bilateral extensor plantar responses. Brain MRI showed acute ischemia in the left hippocampus (Fig. 2a). A follow-up MRI showed contrast-enhancement in the left hippocampus, enhancement of cranial nerve V, VII, VIII, and possible (peri-)vasculitic changes in his BA (Fig. 2b). The patient underwent lumbar puncture which revealed lymphocytic pleocytosis with 55 cells, increased CSF protein of 2.01 g/L, and low glucose of 1.3 mmol/L. Serum IgG borrelia antibody test was positive with a high titer of > 240 AU/mL. Intrathecal AI borrelia test was positive for both IgM and IgG (Table 3). The patient was initially treated with ceftriaxone 4 g IV for three days and doxycycline for 14 days, leading to clinical improvement. However, in August 2017, his gait and speech worsened. CSF analysis showed continuing inflammation (41 leukocytes) and a low CSF glucose level, prompting a new course of doxycycline. MRI revealed no new lesions, and clopidogrel was discontinued in September. A follow-up lumbar puncture in November 2017 showed persistent lymphocytic pleocytosis with 44 leukocytes (Fig. 3).

The patient was readmitted in March 2018 with a stroke in the right pons (Fig. 2b). Lumbar puncture revealed continued inflammation in the CSF and the patient was treated with 14-day IV ceftriaxone therapy and oral prednisolone with tapering. Clopidogrel was reinitiated. In 2019, he was re-admitted with multiple episodes of amnesia and confusion. An electroencephalogram (EEG) revealed focal epileptic activity, and he was started on lamotrigine. DSA in January 2019 was normal and follow-up MRI with contrast in August 2020 revealed no new lesions. CSF had finally normalized by then as well. At his last outpatient visit in February 2023, the patient still suffered from a left hemiparesis but was able to continue his university studies.

#### Case/patient 3

A 69-year-old, previously healthy man, was evaluated at the outpatient neurological clinic in September 2023 with a 30-day history of progressive episodes of short-lasting confusion, aphasia, amnesia, disorientation, and behavioral changes. His wife also reported fatigue, coordination problems and inattention since April 2023. Neurological examination was unremarkable except for disorientation and impaired short-term memory.

Suspecting encephalitis, a lumbar puncture and brain MRI were performed. MRI showed a minor subacute infarction in the right thalamus and chronic infarction in the left thalamus (Supplementary Fig. 1). Lumbar puncture revealed lymphocytic pleocytosis with 136 leukocytes /µL, increased protein of 1.00 g/L and glucose 2.50 mmol/L. Intrathecal and serum *Bb IgG* were above the upper detection limit, preventing the calculation of a *Bb*-AI. *Bb* IgM AI was negative. Extensive tests for other causative agents were negative (Table 3). CXCL-13 levels in the CSF were markedly elevated (> 500 ng/L) but declined rapidly following treatment with IV ceftriaxone (4 g/day). HRVWI-MRI revealed concentric enhancement of the posterior cerebral artery (PCA), BA and VA consistent with CNS vasculitis (Fig. 2c). Prednisolone 100 mg was added with a slow tapering regimen. Two days after treatment initiation, ceftriaxone was substituted for doxycycline, and the patient was discharged.

**Fig. 4:**
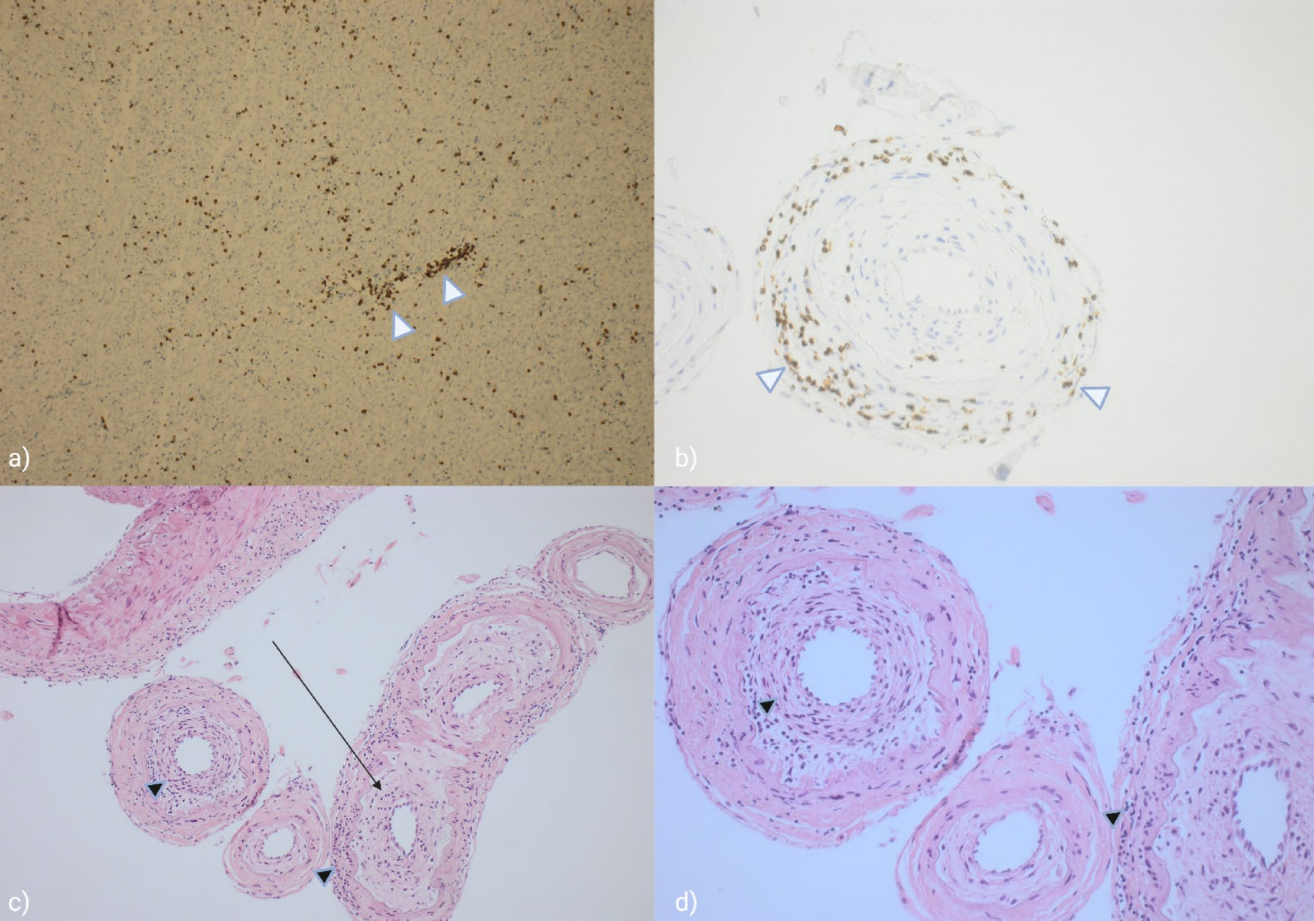
Histopathological examination of the brain biopsy of case 3. **a**, **b** Immunohistochemical CD3 staining showing pronounced inflammation in a cranial nerve and a meningeal blood vessel (**b**) with CD3 positive lymphocytes (white arrowheads). **c**,** d** Pronounced intima fibrosis and hyperplasia (black arrow) and transmural infiltration of meningeal vessels with CD3 positive lymphocytes (black arrowheads)

Three days later, he was readmitted with worsening gait, dysphasia and confusion. MRI revealed a new subacute infarction in the left thalamus. [^18^F]Fluorodeoxyglucose positron emission tomography (¹⁸F-FDG-PET) of the brain was ordered on suspicion of CNS vasculitis (Supplementary Fig. 2). There was no sign of vasculitis, but it showed hypometabolism in the left cerebral hemisphere, basal ganglia and right cerebellar hemisphere as well as hypermetabolism in the left mesial temporal lobe. Despite treatment with methylprednisolone (MP) 1 g iv and antibiotics, the patient’s consciousness worsened into a state of coma. A new MRI revealed acute infarctions in the right thalamus, pons, and cerebellum (Fig. 2d). CTA demonstrated stenosis of the left VA, occlusion of the right PCA, occlusion of the right superior cerebellar artery (SCA), and stenosis of the BA. The patient’s condition continued to decline, and he passed away in early December 2023. A brain autopsy revealed widespread infarctions in the cerebellum, pons, left frontal lobe, occipital lobe, hippocampus, amygdala, hypothalamus, and both thalami. The meninges exhibited lymphocyte infiltrations, dominated by CD3 positive T-cells. Lymphocyte infiltration was also seen in the walls of medium and large meningeal vessels of both veins and arteries (Fig. 4). Arteries showed significant intimal fibrosis with luminal narrowing. These findings were consistent with lymphocytic CNS vasculitis.

## Discussion

### Incidence

The incidence of likely NB-related cerebrovascular events within our NB cohort was 3.1% (13/413) overall and 4.5% for adults (12/267), which is markedly higher than the estimated 0.3-1% in older literature (Winter et al. 2024; Back et al. 2013; Artal 2016). This discrepancy may reflect regional variations, such as a higher prevalence of neuroinvasive *Borrelia* species in our area, but it more likely results from the increased use of high-quality brain and vascular imaging, which enhances diagnostic sensitivity and specificity of CNS vasculitis, the probable cause of these cerebrovascular events. In older NB studies, such as those by Bremell and Dotevall, and a recent systematic review by Knudtzen et al., NB encephalitis was reported as the most common CNS presentation (Knudtzen et al. 2022; Bremell and Dotevall 2014). Notably, the patients in these studies did not consistently undergo advanced imaging that might have revealed vasculitis. For comparison, in our entire NB cohort, only one patient was diagnosed with encephalitis (Rosendahl et al. 2023), whereas older studies reported an incidence of approximately 4–5% (Rauer et al. 2020; Oschmann et al. 1998). Notably, this rate closely matches our observed incidence of NB-associated ischemic stroke and TIA in adults, suggesting that underdiagnosis of vasculitis may have influenced historical data. Supporting this perspective, a recent German case series by Volk et al. identified vasculitis in 5 of 35 consecutive NB patients (14.3%) (Volk et al. 2024). This growing recognition of NB vasculitis aligns with the established pathophysiology of neuroborreliosis, where vasculitic and peri-vasculitic pathology are well-documented as key CNS features (Oksi et al. 1996; Miklossy et al. 1990). Thus, the predominance of vasculitis over encephalitis in modern cohorts may reflect improved diagnostic methodologies rather than a true shift in disease presentation.

In the prospective study by Arboix et al., 1,617 first-ever stroke/TIA patients were registered, of whom 70 (4.3%) were classified as having ischemic stroke of unusual cause (ISUC) (Arboix et al. 2001). Eleven of these 70 cases were attributed to infection, corresponding to 0.68% of all first-ever strokes/TIAs. This confirms that infection-related stroke is rare but recognized. In the present study we believe that NB-related stroke is likely underdiagnosed. Despite nearly half of all NB patients in our total cohort originating from North Zealand Hospital, only one of the 13 NB-associated stroke/TIA cases came from this centre. This disparity strongly suggests that recognition of NB-related vasculitic stroke requires a high index of suspicion, particularly in patients with subacute prodromal symptoms or unexplained infarction patterns.

### Clinical presentation

More than half of our patients presented with gait difficulties, and some exhibited UMN signs, consistent with CNS manifestations of NB such as encephalomyelitis (Bajons et al. 2023; Artal 2016; Volk et al. 2024; Iversen et al. 2023; Dumic et al. 2019; Beuchat et al. 2018; Lindland et al. 2018). These findings are also commonly observed in non-infectious CNS vasculitis and NB-associated vasculitis (Bajons et al. 2023; Artal 2016; Volk et al. 2024; Iversen et al. 2023; Dumic et al. 2019; Lindland et al. 2018; Beuchat et al. 2018). In line with previous studies, approximately half of our patients suffered from a chronic prodromal phase of headache and / or cognitive symptoms prior to stroke (Artal 2016; Topakian et al. 2008; Back and Grünig 2016; Arboix et al. 2001). The symptoms of cases 3, 4, 6, 11, and 13 evolved insidiously, and the detection of acute diffusion-restricted lesions on MRI was unexpected. This spectrum mirrors primary angiitis of the central nervous system (PACNS), where initial stroke symptoms are reported in 11.5–51% across cohorts, lowest in biopsy-confirmed small-vessel disease (11–22%) and highest in large/medium-vessel PACNS (62%, LV-PACNS), and where walking disability, headache, and cognitive decline are frequent presenting or evolving features (Reddy et al. 2025; Sundaram et al. 2019; Paramasivan et al. 2024).

Notably, only patients 2 and 12 (Table 2) presented with acute stroke symptoms as the initial manifestation. Patient 7 (Table 2) initially exhibited acute 2-hour lasting focal neurological symptoms, and only after admission did her mother vaguely recall mild prodromal symptoms prior to admission.

These findings highlight the importance of actively inquiring about prodromal symptoms, particularly in cases of stroke without conventional vascular risk factors. The presence of UMN signs that cannot be explained by an acute stroke should prompt consideration of alternative stroke etiologies. Clinicians should also inquire about a history of rash or tick bites, as half of our patients reported one or both. However, it is important to note the variability in the literature regarding the frequency of prodromal symptoms and tick exposure preceding stroke in NB-associated vasculitis (Garkowski et al. 2017; Back et al. 2013).

### Cerebrospinal fluid presentation

CSF analysis revealed lymphocytic pleocytosis in all but two of the 13 patients, with a median leukocyte count of 105/µL (range 1–832). This finding aligns with previous studies on NB vasculitis and did not differ significantly from the rest of the cohort (*p* = 0.83) (Garkowski et al. 2017; Winter et al. 2024). Pleocytosis is uncommon in ischemic stroke without inflammatory or infectious etiology (Rundblad et al. 2023).

CSF glucose levels were significantly lower in NB patients with cerebrovascular manifestations compared to the rest of the cohort. This may reflect more profound CNS inflammation, possibly driven by more pathogenic *Bb* species and/or longer symptom duration prior to diagnosis. However, we found no relationship between symptom duration and CSF WBC in these patients.

Consistent with Volk et al., Q-alb and IgG index, markers of blood-brain barrier disruption, were elevated and higher than the rest of the cohort (Volk et al. 2024).

Case 2 (Tables 1, 2, 3) highlights that CSF inflammation can persist for years after treatment, with strokes occurring months or even years later (Fig. 3). This may result from a combination of persistent vessel stenosis, scarring, and ongoing, albeit diminishing, inflammation. Case 5 (Tables 1, 2, 3) presented with acrodermatitis chronica atrophicans (ACA)-like rash 6 months before her first stroke and went on to experience two additional strokes over the subsequent 2 years. Despite biopsy-confirmed ACA, and a markedly elevated *Bb* IgG IT index, her CSF lacked pleocytosis. This may partly be explained by a 17-day course of penicillin prior to the CSF examination (Krogen et al. 2022). Furthermore, previous PCR and culture studies have demonstrated that *Bbsl* can be found in the CSF even in the absence of pleocytosis (Strle et al. 2006). This phenomenon is particularly notable in *B. afzelii*-associated NB, where CSF WBC appear to decrease as the disease progresses (Strle et al. 2006; Rožič et al. 2019; Busch et al. 1996; Ornstein et al. 2002). Non-infectious CNS vasculitis can present with normal CSF, and rare cases of NB vasculitis without pleocytosis have been described in the literature (Garkowski et al. 2017; Beuker et al. 2021; Riescher et al. 2023).

### Imaging and stroke location

As in other reports of NB-associated ischemic strokes, the posterior circulation in our cohort was more commonly affected (9/13, 69%) compared with typical ischemic strokes, where posterior circulation strokes account for 20–25% of the total amount (Garkowski et al. 2017; Winter et al. 2024; Demir Unal 2023). This pattern is also observed in other unusual causes of ischemic stroke (Arboix et al. 2001).

While brain inflammation can produce a hyperintense signal on DWI, all patients in our case series who were classified as having MRI evidence of brain infarction, had hyperintense lesions on DWI with a characteristic appearance—appropriate location and ADC-confirmed diffusion restriction—consistent with acute or subacute ischemic stroke. Furthermore, 8/11 patients with DWI changes had follow-up MRIs and stroke was confirmed in all eight patients (Cases 2–6, 8, 12, and 13, Table 2).

The gold standard for diagnosing CNS vasculitis is DSA and meningocortical biopsy (MCB), yet their diagnostic yield is only in the 70–88% and 60–70% range, respectively (Sundaram et al. 2021). A promising alternative is HRVWI-MRI, a novel non-invasive technique for early detection of CNS vasculitis (Sundaram et al. 2021). In a study by Sundaram et al., a neuroradiologist correctly identified vessel wall abnormalities in 20 out of 21 patients with PACNS based on HRVWI-MRI (Sundaram et al. 2021). Another study demonstrated that HRVWI-MRI could detect medium-sized vessel CNS vasculitis with a specificity of 95% and sensitivity of 94% (Ferlini et al. 2022). Moreover, in direct comparisons with DSA, HRVWI-MRI has shown higher, albeit not statistically significant, sensitivity in diagnosing CNS vasculitis (Park et al. 2017).

In this study, HRVWI-MRI demonstrated vascular abnormalities consistent with vasculitis in all three patients in whom it was performed. These findings align with results from Winter et al., where vascular concentric contrast enhancement was observed in all 40 patients examined (Winter et al. 2024).

Evidence on whether HRVWI-MRI can differentiate between perivascular inflammation in meningitis or inflammation of the vessel wall itself, is limited to small series and case reports. Importantly, our case 3 did show that vessel-wall enhancement with wall thickening on HR-VWI (Fig. 2c) corresponded at autopsy to a lymphocytic transmural vasculitis with significant structural vessel-wall changes (notably severe intimal hyperplasia). A case study by van Rooij et al. showed similar findings; however, their case 2 had positive HR-VWI but a negative biopsy despite compelling evidence for LV-PACNS (Van Rooij et al. 2018). Similarly, Zeiler et al. used HR-VWI to target brain/meningeal biopsies in suspected CNS vasculitis and found vascular inflammation in 8/9 biopsies; however, 3/8 showed only perivascular inflammation, suggesting that HR-VWI can overestimate true mural vasculitis (Zeiler et al. 2018). These findings are not surprising since large arteries with concentric enhancement were not biopsied; instead, more superficial linear areas of enhancement corresponding to leptomeningeal venules were sampled. Consistent with these sampling limitations, a meta-analysis by Beuker et al. found diagnostic biopsies in only ~ 8% of angiogram-confirmed LV-PACNS cases (Beuker et al. 2021).

Since HRVWI-MRI has only been available at our hospital since 2021, it was not performed on a larger number of patients. Nevertheless, given its non-invasive nature and seemingly high sensitivity, HRVWI-MRI may be considered as part of the diagnostic workup in patients with suspected NB vasculitis.

Despite multiple strokes, the ^18^F-FDG-PET of case 3 showed no sign of vasculitis. Although whole-body ¹⁸F-FDG-PET has high sensitivity in systemic vasculitis, the modality has not been systematically evaluated in NB-vasculitis or PACNS (Deb-Chatterji et al. 2025). In an unselected NB cohort (*n* = 23), most patients exhibited temporal hypometabolism and in some cases diffuse cortical hypometabolism, consistent with our case and with additional NB-vasculitis cases diagnosed at our institution after that study period (Newberg et al. 2002). In a 2025 series of 20 PACNS patients, all 18 examined were reported as PET-negative, although it was not specified how many underwent brain ¹⁸F-FDG-PET (Scoppettuolo et al. 2025). Conversely, and similar to our case 3, biopsy-proven granulomatous PACNS has been shown to exhibit hemispheric cortical hypometabolism on brain ¹⁸F-FDG-PET (Sagnelli et al. 2024). A likely explanation is that affected vessel calibers in CNS vasculitis are below the spatial resolution of ¹⁸F-FDG-PET and that detection is further challenged by high physiologic cortical background metabolism. Consequently, in CNS vasculitis, PET is mainly applied to exclude mimics (encephalitis, lymphoma, cancer, systemic vasculitis) rather than to confirm the vasculitis itself. Nevertheless, we believe that our PET findings provide mechanistic clues and may contribute to a better understanding of the complex pathophysiology in NB-vasculitis.

Other potential imaging findings of interest include cerebral white matter lesions (WML) and their possible link to NB-related cerebrovascular events. Although most studies have not found a link between WML and NB, a recent Norwegian longitudinal MRI study using quantitative white matter hyperintensities (WMHs) volumetry found a small reduction in WMH volume in treated NB patients over follow-up, whereas controls showed a slight increase (Andreassen et al. 2021; Aalto et al. 2007; Lindland et al. 2025). Yet, most published MRI series include all NB patients rather than only those with CNS involvement or late NB, which probably dilutes detection of NB-vasculitis–specific patterns. In several cerebral small vessel disease (CSVD) studies, diffusion tensor imaging (DTI) detected microstructural injury even in normal-appearing white matter on FLAIR and often preceded or exceeded visible WML progression (O’Sullivan et al. 2001; Maillard et al. 2011; De Groot et al. 2013; Tuladhar et al. 2015; Segura et al. 2010). On this basis, DTI may represent a more sensitive imaging method for detecting subtle NB-related microvascular or inflammatory injury, particularly in NB-associated vasculitis. To date, however, no systematic DTI studies have been performed in NB patients who have CNS manifestations and fulfill the EFNS criteria, representing a relevant future research avenue.

### Treatment

The EFNS guidelines recommend either 14 days of IV ceftriaxone 2 g daily or oral doxycycline 200 mg daily therapy as treatment of NB vasculitis (Rauer et al. 2020; Mygland et al. 2010). This approach leads to complete recovery in 75% of cases, an incomplete but stable response with residual neurological sequelae in about 20%, and fatality in few cases despite appropriate therapy (4.7%) (Garkowski et al. 2017). Five out of thirteen of our patients were initially treated with high doses of ceftriaxone (4 g daily) while they awaited Intrathecal *Bb* tests and other causative agents were still being considered.

The progressive nature of NB vasculitis, even with treatment, was demonstrated by Winter et al., where modified Rankin scale (mRS) remained unchanged and NIH stroke scale scores (NIHSS) had worsened 1 year after admission (Winter et al. 2024). Both mRS and NIHSS were significantly worse at follow-up for NB vasculitis patients compared to matched stroke patients despite relevant antibiotic treatment (Winter et al. 2024). Some authors advocate adjunctive corticosteroids and prophylactic low-dose aspirin therapy although there is no high-level evidence supporting their benefit (Rauer et al. 2020). Importantly, in the study by Winter et al., no association was found between the type or duration of antibiotic or steroid therapy and clinical outcomes (Winter et al. 2024). We believe this progressive course (resembling PACNS) reflects prolonged inflammation and structural changes (stenosis) to vessel walls. In cases 1 and 4, follow-up HR-VWI demonstrated persistent, although gradually diminishing, vessel wall enhancement that took approximately 1 year to resolve, supporting a protracted inflammatory/structural course. Case 3 (Tables 1, 2, 3) ended in fatality despite relevant treatment with ceftriaxone, doxycycline, and MP/prednisolone. It could be argued that cyclophosphamide might have been considered as an adjunct therapy in this case. However, like corticosteroids, there is no randomized evidence supporting its use in this context. The available data is limited to rare case reports, which carry a significant risk of publication bias favoring successful outcomes (Bajons et al. 2023; Rauer et al. 2020; Komdeur 2001).

### Neuropathology

NB vasculitis occurs due to inflammation in the meninges that extends into cerebral arteries, ultimately leading to vessel narrowing and thrombosis (Artal 2016). This mechanism closely resembles meningovascular neurosyphilis, an important differential diagnosis, where obliteration and thrombosis of the vessel lumen similarly results from an initial chronic meningitis (Miklossy et al. 1990). The brain autopsy of our patient (case 3, see Tables 1, 2, 3; Fig. 4) showed lymphocytic infiltration of the meninges, consistent with chronic meningitis and lymphocytic infiltration into the meningeal and parenchymal vessels, resembling the histopathological patterns of lymphocytic biopsy-proven PACNS (Jennette et al. 2013). A similar finding was reported in a 1990 case study of a fatal NB vasculitis, where brain autopsy revealed lymphocytic vascular infiltration, fibrosis, and luminal narrowing (Miklossy et al. 1990). Furthermore, a case series analyzing biopsies from NB vasculitis patients demonstrated a combination of vascular and perivascular inflammation, characterized by lymphocytic infiltration within and surrounding vessel walls (Oksi et al. 1996).

### Strengths and limitations

While most data on NB-associated vasculitis and ischemic stroke is limited to singular case reports, systematic reviews thereof, and small cohort studies (Garkowski et al. 2017; Bajons et al. 2023; Zajkowska et al. 2015; Winter et al. 2024; Miklossy et al. 1990; Back et al. 2013; Topakian et al. 2008; Akkurt et al. 2023; Komdeur 2001), this study provides valuable insights by including a relatively large number of NB-associated stroke and TIA patients within a well-defined cohort from an endemic area in Denmark. Additionally, comparison of CSF findings between NB patients with cerebrovascular manifestations and the broader NB cohort provides a deeper understanding of the disease’s characteristics. Furthermore, the inclusion of a brain biopsy—rarely reported in NB vasculitis literature—adds further weight to the findings presented here (Oksi et al. 1996; Miklossy et al. 1990).

A key limitation of this study is its retrospective nature, which resulted in variability in the investigation, reporting of symptoms, treatment, and follow-up of patients. Follow-up duration differed greatly, complicating the evaluation of treatment outcomes. For instance, while the first patient has been followed continuously since her presentation in 2022, patient 10 did not receive any follow-up after discharge for his stroke and NB diagnosis (Table 1).

**Fig. 5:**
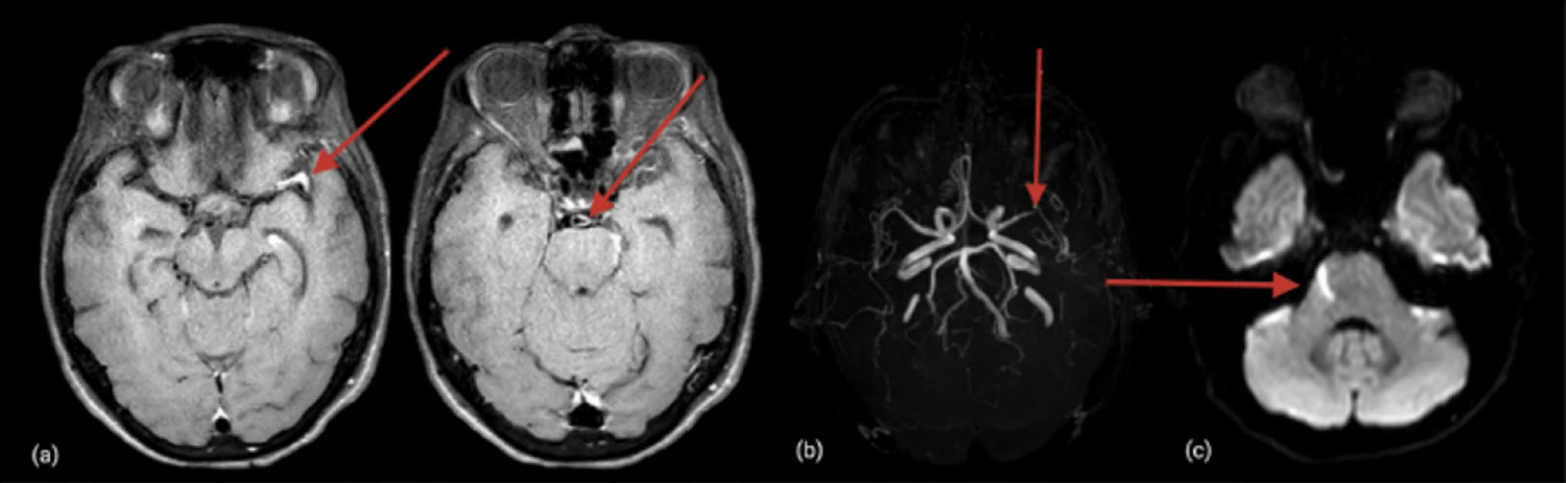
MRI of the cerebrum, case 4, 2024. See Tables 1, 2, 3 for case 4. **a** HRVWI-MRI with concentric enhancement of the BA, left SCA, left M1 and M2 branches of the MCA (red arrows). **b** MRI-TOF with stenosis of the left M1 and M2 branches of the MCA (red arrow). **c** subacute pontine infarction on the right side (red arrow). HRVWI-MRI (MRI High resolution vessel wall imaging), BA (basilar artery), SCA (superior cerebellar artery), MCA (middle cerebral artery), TOF (time of flight)

The diagnostic investigations for some patients were also incomplete. Patient 9 (Tables 1, 2, 3) did not undergo DSA, CTA, MR-angiography, or HRVWI-MRI, which complicated the confirmation of NB vasculitis or exclusion of other causes for stroke. Similarly, patient 10 (Tables 1, 2, 3), despite being young and without conventional stroke risk factors, did not receive a TTE to exclude PFO. Furthermore, no efforts were made to screen for infectious agents other than *Bb*. Nevertheless, given the proximity of his NB and stroke diagnoses (2 months apart), the classical symptom presentation (neck and shoulder pain, dysesthesia, and facial paralysis), and the absence of other risk factors, NB-associated ischemic stroke remains the most probable diagnosis (Fig. 5).

## Conclusion

Although we found a higher incidence of cerebrovascular manifestations in NB patients compared to older studies, stroke related to NB remains a rare cause overall, as our cases only accounted for 0.1% of total strokes in our region, similar to previous reports (Mironova et al. 2021). Thus, like primary CNS vasculitis, diagnosing this potentially treatable but rare cause of stroke requires a high degree of suspicion.

Prodromal symptoms, unexplained clinical findings, multiple-territory infarctions, unexplained intracranial vessel stenosis or occlusions, vessel wall enhancement on HRVWI-MRI, meningeal or cranial nerve enhancement, and potential tick exposure should prompt clinicians to consider NB as an underlying cause of stroke or TIA in endemic areas.

While antibiotic therapy often leads to favorable outcomes, some patients suffer persistent neurological sequelae or even fatal courses despite appropriate treatment. More systematic research is required to determine optimal antibiotic regimens and explore the potential role of adjunctive immunosuppressive therapies in improving outcomes for these challenging cases.

The most important next step is the establishment of prospective studies in unselected stroke/TIA patients that are specifically designed to identify infectious vasculitic mechanisms to more accurately determine the true incidence of NB-related cerebrovascular events.

In a broader perspective, future studies should also explore advanced imaging markers of cerebral small vessel disease, including DTI–based measures of microstructural injury, to improve detection of subtle NB-related vasculitic or microvascular involvement beyond what is visible on conventional MRI. This remains an unexplored but promising direction for refining NB diagnostics.

## Supplementary Information

Below is the link to the electronic supplementary material.

Supplementary Material 1

## Data Availability

No datasets were generated or analysed during the current study.
