# Supplementary material for: Ischemic stroke and transient ischemic attack associated with neuroborreliosis: a Danish retrospective cohort
Source: J Neural Transm (Vienna). 2026 Jan 16;133(7):1553–71. doi: 10.1007/s00702-025-03087-8 (PMC13428788; doi:10.1007/s00702-025-03087-8)
Supplement: Supplementary file 1 — Supplementary Material 1 [file 702_2025_3087_MOESM1_ESM.docx]

**Supplementary figures and tables.**

***Supplementary figure 1:*** *Chronological MRI of the cerebrum (without contrast) with ischemic lesions of case 3*

***
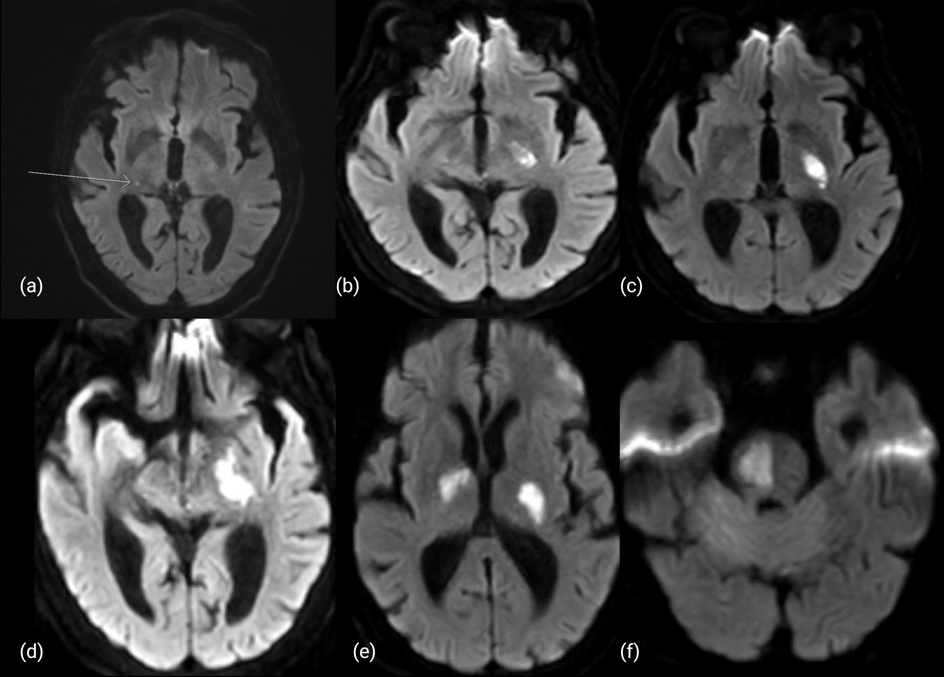
***

1a: DWI showing a small subacute ischemic infarction in the right thalamus (white arrow), 1b: acute infarction with DWI lesion in the left thalamus 18 days from first MRI, 2c-d: evolving ischemic changes with DWI lesions in the left thalamus, basal ganglia and mesial temporal lobe 21 days from first MRI, 1e-f: acute infarction with DWI lesions in the right thalamus, pons and cerebellum 25 days from the first MRI Abbreviations: MRI (magnetic resonance imaging), DWI (diffusion-weighted magnetic resonance imaging**)**.

***Supplementary figure 2:*** *F-18-FDG PET scan of the brain, case 3*


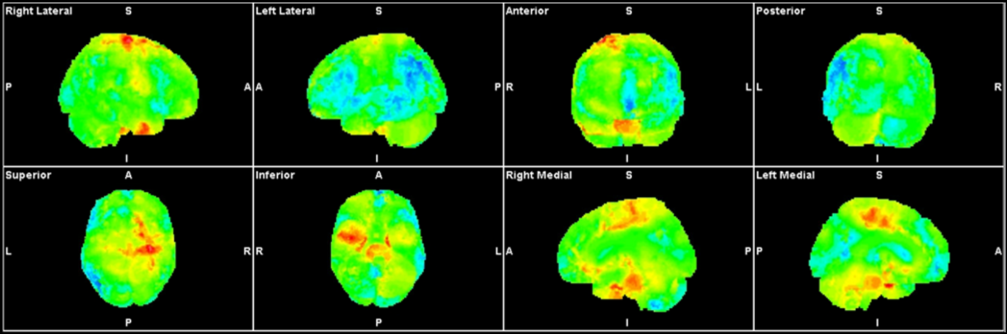
hypometabolism in the left cerebral hemisphere, basal ganglia and right cerebellar hemisphere as well as hypermetabolism in the left mesial temporal lobe.

|  | Adults without vasculitis | Only vasculitis  Patients | p-value  (Adults without vasculitis vs vasculitis | ^*^Total observations adults without vasculitis | ^*^Total observations only vasculitis |
| --- | --- | --- | --- | --- | --- |
| Median WBC count (range) (ref: 0-4) /µL | 78 (0-1350) | 105 (1-832) | 0.83 | 253/255 | 13/13 |
| Median WBC mononuclear cell percentage (range) | 99 (55-100) | 97 (68-100) | 0.21 | 221/255 | 11/13 |
| Mean Protein level±SD (ref: 0.2-0.6) g/L | 1.24±0.77 | 2.12±1.48 | 0.055 | 247/255 | 13/13 |
| Mean CSF/blood Glucose±SD (ref: 0.48-0.87) | 0.53±0.12 | 0.38±0.16 | 0.007 | 202/255 | 12/13 |
| Median Q-Alb (range) (ref: <6.3) | 16.58 (2.7-72.00) | 27.02 (5.35-45.10) | 0.346 | 51/255 | 8/13 |
| Mean IgG index±SD (ref: <0.63) | 0.77±0.42 | 1.354±0.88 | 0.109 | 41/255 | 8/13 |
| Mean glucose±SD (ref: 2.8-4.4) g/L | 3.4±0.94 | 2.11±0.95 | <0.001 | 245/255 | 13/13 |

***Supplementary table 1:*** *Cerebrospinal fluid findings at admission in patients with neuroborreliosis with and without vasculitis from the Capital region of Denmark, January 2016 – January 2024.*

Continuous variables compared with independent two sample t-test. Abbreviations: NB (Lyme neuroborreliosis), Q-Alb (albumin quotient), IgG index (immunoglobulin G index), ref (reference). ^*^Number of patients with available data.
